# Dengue virus is particularly sensitive to interference with long-chain fatty acid elongation and desaturation

**DOI:** 10.1016/j.jbc.2025.108222

**Published:** 2025-01-23

**Authors:** Julia Hehner, Lisa Ludenia, Laura Bierau, Anja Schöbel, Martin Schauflinger, Yvonne F. Grande, Dominik Schwudke, Eva Herker

**Affiliations:** 1 Institute of Virology, Philipps University Marburg, Marburg, Germany; 2 Division of Bioanalytical Chemistry, Research Center Borstel - Leibniz Lung Center, Borstel, Germany; 3 German Center for Infection Research, Thematic Translational Unit Tuberculosis, Partner Site Hamburg-Lübeck-Borstel-Riems, Borstel, Germany; 4 German Center for Lung Research (DZL), Airway Research Center North (ARCN), Research Center Borstel, Leibniz Lung Center, Borstel, Germany

**Keywords:** virus, lipid, polyunsaturated fatty acid (PUFA), flavivirus, fatty acid metabolism, host–pathogen interaction

## Abstract

Orthoflaviviruses are emerging arthropod-borne pathogens whose replication cycle is tightly linked to host lipid metabolism. Previous lipidomic studies demonstrated that infection with the closely related hepatitis C virus (HCV) changes the fatty acid (FA) profile of several lipid classes. Lipids in HCV-infected cells had more very long-chain and desaturated FAs and viral replication relied on functional FA elongation and desaturation. Here, we systematically analyzed the role of FA elongases and desaturases in infection models of the most prevalent pathogenic orthoflaviviruses, dengue (DENV), Zika (ZIKV), West Nile (WNV), yellow fever (YFV), and tick-borne encephalitis virus (TBEV). Knockdown of desaturases and elongases in Huh7 cells only marginally affected ZIKV, WNV, YFV, and TBEV replication, while DENV titers were strongly reduced. This was most prominent for enzymes involved in very long–chain fatty acid synthesis. In detail, knockdown of the FA elongase ELOVL4, which catalyzes ultra-long-chain FA synthesis, significantly reduced DENV titers, decreased the formation of replication intermediates, and lowered viral protein levels in DENV-infected hepatoma cells, suggesting a function of ELOVL4 in DENV RNA replication. In contrast, the activity of FA desaturase FADS2, rate-limiting in poly-unsaturated FA biosynthesis, is not involved in viral RNA replication or translation, but is essentially required for the formation of infectious DENV particles. Further, in immunocompetent immortalized microglial cells, FADS2 deletion additionally limits viral replication through increased expression of interferon-stimulated genes in response to DENV infection. Taken together, enzymes involved in very long–chain FA synthesis are critical for different steps of DENV replication.

With over 100 million confirmed cases every year, dengue virus (DENV) is the most prevalent arthropod-borne viral pathogen. It presents a major public health concern, as DENV infections are associated with significant morbidity, mortality, and high economic costs, particularly in developing countries (1). Endemic in tropical and subtropical regions, DENV is primarily transmitted by mosquitos of the species *Aedes aegypti* and occurs in four distinct but closely related serotypes (DENV-1, DENV-2, DENV-3, and DENV-4), all infectious for humans (2, 3). Primary DENV infections are asymptomatic in 80% of cases. A symptomatic manifestation can range from mild symptoms such as headache, mild fever, and muscle pain, as well as nausea and vomiting, to the development of life-threatening dengue hemorrhagic fever (DHF) and dengue shock syndrome (DSS) (4). A heterotypic secondary DENV infection increases the risk for severe dengue, most likely resulting from antibody-dependent enhancement (ADE) (5, 6). While there are two currently licensed vaccines available (2019), a specific antiviral treatment for DENV has yet to be developed.

Within the Flaviviridae family, DENV belongs to the genus *orthoflavivirus*, which also comprises other important human pathogens such as yellow fever virus (YFV), Zika virus (ZIKV), West Nile virus (WNV), and tick-borne encephalitis virus (TBEV) (1, 7, 8, 9). Orthoflaviviruses are enveloped positive-strand RNA viruses with a 10 to 11 kb single-stranded genome containing one open reading frame (ORF) flanked by 5′ and 3′ untranslated regions (UTR). Cleavage of the encoded single polyprotein by host and viral proteases gives rise to the three structural (capsid [C], precursor membrane [prM], and envelope [E]) and seven non-structural proteins (NS1, NS2A, NS2B, NS3, NS4A, NS4B, and NS5) (1). Orthoflaviviruses enter the cells *via* endocytosis, where several host cell membrane factors are involved in the attachment and binding of the structural proteins. RNA replication occurs at the endoplasmic reticulum (ER), where orthoflaviviruses induce membrane invaginations, leading to the formation of clusters of vesicle packets (VPs) that represent viral replication organelles (ROs) (8). These ROs contain all non-structural proteins except NS1, which is present in the ER lumen, as well as double-stranded RNA (dsRNA) (8, 10, 11). Following the assembly of virions at the ER, the particles are transported into the Golgi apparatus for maturation and are subsequently released through the secretory pathway.

Concomitant with the changes in membrane structure, flavivirus infection induces profound alterations of the host lipid profile (12, 13). Previous studies indicate that human pathogenic flaviviruses hijack lipid metabolic pathways (14). For example, fatty acid synthase (FASN) interacts with NS3 and is translocated to viral ROs (15). Furthermore, flaviviruses are known to modulate fatty acids (FAs) and cholesterol biosynthesis (16). FAs are components of lipids such as triglycerides (TAG), cholesterol esters (CE), and phospholipids (PL) and can be divided into saturated FAs (SFA) and unsaturated fatty acids like monounsaturated FAs (MUFAs) and polyunsaturated FAs (PUFAs) (17). The extent of fatty acid chain length and degree of desaturation depends on the enzymatic activity of elongases and desaturases and can directly impact membrane curvature and fluidity (18). Fatty acid elongases interact with 3-ketoacyl-CoA reductase, a dehydratase, and trans-2,3-enoyl-CoA reductase to elongate FAs (19). The reaction step catalyzed by the elongases is both rate-limiting and confers substrate specificity. In addition, FA desaturases (FADS and SCD enzymes) introduce double bonds into fatty acyl chains, catalyzing the biosynthesis of MUFAs and PUFAs (19, 20).

Stearyl-CoA desaturase 1 (SCD), the enzyme that initiates the formation of MUFAs, was found to be required for viral genome replication of DENV-2 (21) as well as HCV (22). Additionally, inhibition of SCD was shown to reduce HCV viral replication (23). Interestingly, HCV-infected cells showed a higher abundance of long-chain fatty acids with more double bonds (23). Knockdown of PUFA-synthesizing enzymes impaired HCV progeny production, demonstrating that PUFAs are required for HCV replication.

Finally, the lipid composition of the viral envelope is a determinant of virion infectivity. Depletion of cholesterol from the viral envelope leads to a reduction in DENV infectivity (24), whereas the incorporation of certain lipids, especially phosphatidylserine, enables viral entry into the host cell by exploiting apoptotic mimicry (25, 26).

Here, we systematically analyzed the role of FA elongases and desaturases in cell-culture infection models of the most prevalent human pathogenic orthoflaviviruses using RNAi. DENV was most sensitive towards impaired FA elongation and desaturation. As the strongest effects were observed for FADS2 and ELOVL4, which are important for PUFA and ultra-long-chain FA (ULCFA) synthesis, we analyzed those two enzymes in greater detail. While ELOVL4 knockdown impacts viral RO formation, FADS2 expression and activity are important for the production of infectious particles. In addition, FADS2 deletion limits viral replication in immunocompetent cells through increased expression of interferon-stimulated genes (ISGs) in response to DENV infection.

## Results

### DENV replication is particularly sensitive to depletion of enzymes involved in long-chain fatty acid elongation and desaturation

**Figure 1:**
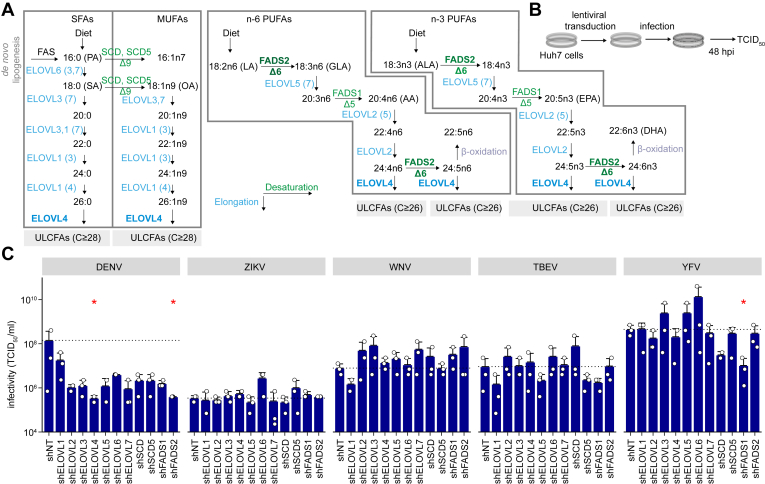
**DENV replication is sensitive to the depletion of enzymes involved in fatty acid elongation and desaturation.***A*, scheme of saturated (SFA), monounsaturated (MUFA), and polyunsaturated fatty acid (PUFA) biosynthesis indicating the different elongation (catalyzed by elongases, ELOVL, *blue*) and desaturation (desaturases SCD, FADS, *green*) steps. *B*, Huh7 cells were transduced with lentiviruses carrying shRNAs targeting different FA elongases and desaturases followed by orthoflavivirus infection. *C*, at 4 days post transduction (dpt), cells were infected with different orthoflaviviruses (ZIKV MOI 0.1, WNV MOI 0.001, TBEV MOI 0.5, YFV MOI 0.005, DENV MOI 0.05). Supernatants were harvested at 48 h post-infection (hpi) and infectivity was determined *via* TCID_50_ titration (Mean ± SEM + SD, n = 3, ∗*p* ≤ 0.05, Mann-Whitney *U* Test).

The synthesis of MUFAs and PUFAs (Fig. 1*A*) involves seven different FA elongases (ELOVL1–7) that add two carbons to the elongated acyl chain, as well as FA desaturases (FADS1 and 2, as well as SCD and SCD5). To investigate if FA elongation and desaturation are needed for orthoflavivirus infection, we specifically depleted these enzymes in Huh7 cells using a lentiviral shRNA delivery system (23). The respective Huh7 knockdown cells were infected with viral stocks of DENV, ZIKV, WNV, TBEV, and YFV-17D, and viral titers were determined 48 h post-infection (hpi) (Fig. 1, *B* and *C*). DENV titers were drastically reduced in almost all elongase- and desaturase-knockdown cells (Fig. 1*C*). Only the depletion of FADS6 did not affect DENV titers. Among all elongases and desaturases investigated in this study, the downregulation of FADS2 and ELOVL4 most strongly and significantly decreased DENV titers.

In contrast, ZIKV and YFV infectivity in culture supernatants remained unchanged in all elongase- and desaturase-knockdown cells. Only a slight reduction of TBEV titers was observed in shELOVL1-, shELOVL5-, shFADS1-, and shSCD5-expressing cells. While depletion of ELOVL1 also slightly decreased WNV titers, knockdown of the other elongases and desaturases did not alter viral titers or even marginally increase them (Fig. 1*C*).

Therefore, our results indicate that FADS2 and ELOVL4 enzymes or their reaction products are especially important for DENV replication. FADS2 catalyzes the rate-limiting step in PUFA synthesis while ELOVL4 catalyzes ultra-long-chain fatty acid (ULCFA) biosynthesis (Fig. 1*A*).

### Dengue virus infection alters the abundance of long-chain fatty acids

**Figure 2:**
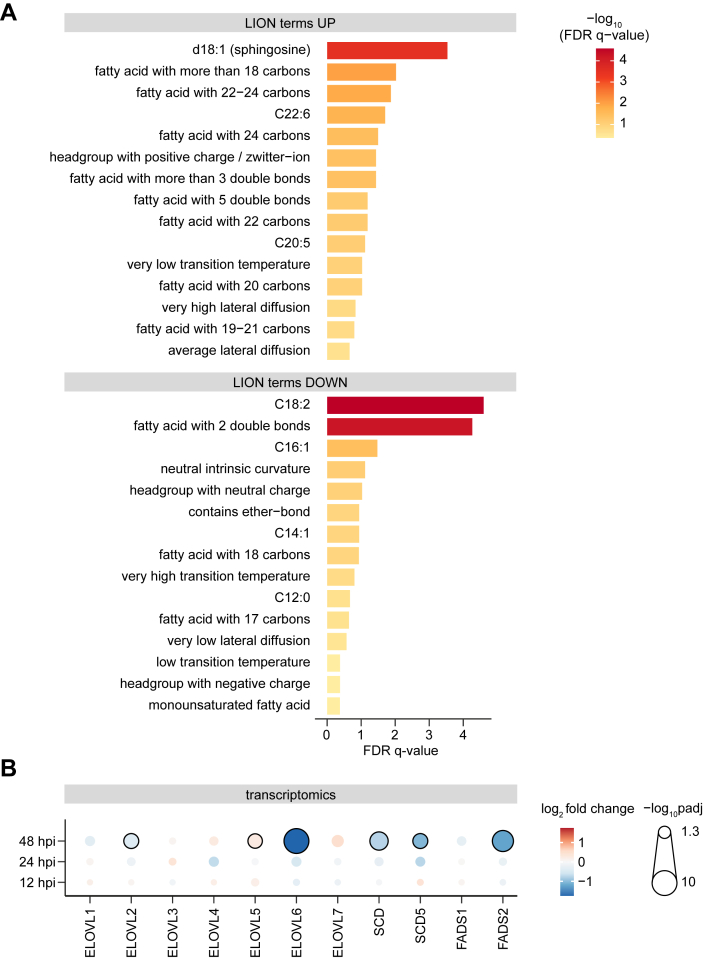
**Lipid species containing very long–chain fatty acids with double bonds are more abundant in cells infected with DENV.***A*, analysis of lipidomic datasets from Huh7 cells infected with DENV for 48 h. Lipid ontology analysis of lipid species abundance per μg protein using the Lipid Ontology Enrichment Analysis Web application (LION/web). The top up- and downregulated lipid ontology terms, selected for physical and chemical properties with fatty acid prediction of DENV-infected cells compared to mock controls are shown with the color code indicating the -log_10_ FDR adjusted *q*-values. *B*, Log_2_ fold-change of elongase and desaturase mRNA expression level determined by transcriptome analysis of the same samples. Shown is the average of 3 independent experiments, with *red/blue* representing increases/decreases and *grey* indicating NA. FDR-adjusted *p*-values are indicated by point size. Significant values are indicated with *black outlines*.

In our previous lipidomic study, we compared orthoflavivirus-infected cells to uninfected controls (12) (dataset available *via* LipidCompass (accession number LCE00000015) or Metabolights (accession number MTBLS11030)). Using this dataset, we further performed lipid ontology (LION) enrichment analysis *via* the web application LION/web (Fig. 2*A*). We preselected the LION-terms “physical and chemical properties” and predicted FA association and used the dataset DENV-infected cells vs mock controls at 48 hpi (Table S1) Comparing the physical and chemical properties of the altered lipids, we discovered that DENV-infected cells had a higher abundance of FAs with more than 18 carbons up to 24 carbons compared to uninfected cells (Fig. 2*A* and Table S2). Additionally, FAs with 3 or 5 double bonds were more abundant whereas FAs with 2 double bonds or MUFAs were decreased in DENV-infected cells. We then analyzed the corresponding transcriptomic data (12) (dataset available *via* the Gene Expression Omnibus database (accession code GSE264306) and specifically examined the transcript levels of elongases and desaturases in DENV infection (Fig. 2*B*). Early in infection (12 hpi), we only observed minor differences in the expression levels for all analyzed genes. As the infection progressed, *FADS2* as well as *SCD* mRNAs were both significantly reduced (48 hpi). We also observed that *ELOVL4* expression slightly but gradually increased during infection.

### Knockdown of ELOVL4 and FADS2 impacts different steps in the DENV replication cycle

**Figure 3:**
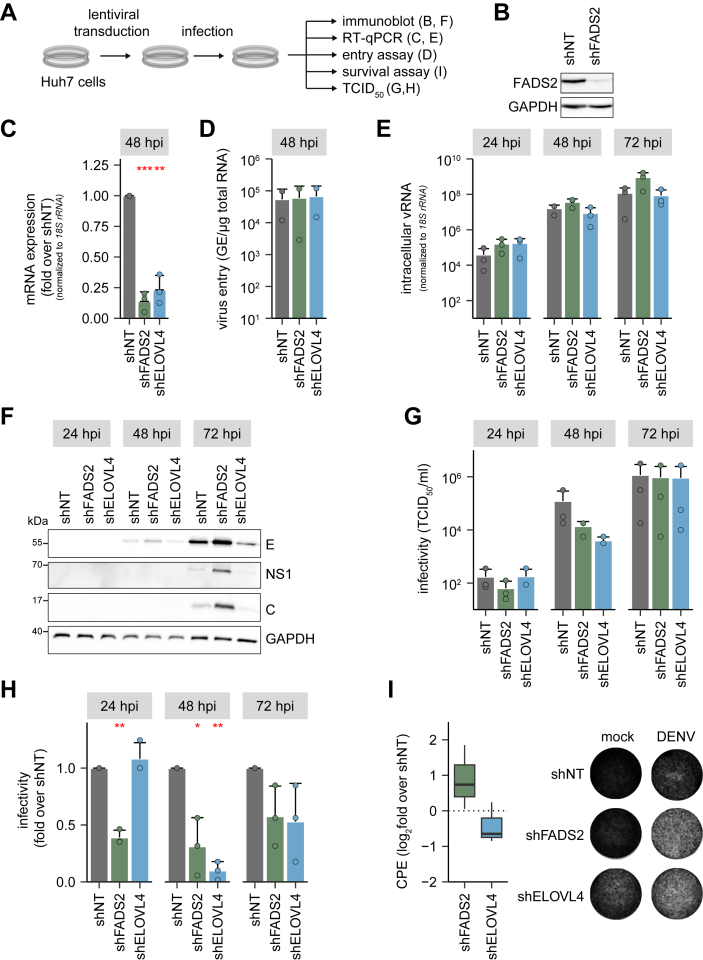
**Knockdown of FADS2 and ELOVL4 both reduce DENV infectious titers but impact different virus replication steps.***A*, scheme of the experiment: Huh7 cells were transduced with lentiviral particles expressing shFADS2 or shELOVL4. shNT served as control. Four dpt, cells were infected with DENV (MOI 0.5) and 24, 48, and 72 hpi supernatants were harvested and cells were lysed for protein and RNA extraction, as well as determination of viral titers. *B*, knockdown of FADS2 was confirmed by immunoblotting. *C*, knockdown efficacy of FADS and ELOVL4 was validated by RT-qPCR (Mean + SD, n = 3, ∗∗*p* ≤ 0.01, ∗∗*p* ≤ 0.001, one sample Student’s *t* test). *D*, viral entry was determined by measuring viral genome equivalents (GE) 4 h post-infection of Huh7 cells (MOI 5) using RT-qPCR (Mean + SD, 2 independent experiments. *E*, intracellular virus genome copies were measured *via* RT-qPCR. Shown are virus genome equivalents (GE) per μg total cellular RNA normalized to *18S rRNA* (Mean + SD, n = 3). *F*, intracellular levels of viral E, NS1, and DENV C protein were assessed by immunoblotting at indicated time points. Shown is one representative immunoblot (n = 3). *G*, infectivity was calculated by TCID_50_ titration. *H*, infectivity fold over shNT, calculated by TCID_50_ titration (Mean + SD, n = 3, ∗*p* ≤ 0.05, ∗∗*p* ≤ 0.01, one sample Student’s *t* test). *I*, transduced cells were infected with DENV (MOI 0.5) for 72 h, fixed, and stained with *crystal violet*. Calculation of CPE was performed using the mean *grey* intensity quantified in Fiji. Box- and whisker plot indicates CPE as log_2_ fold change over shNT (*center line*: median, box limits: *upper and lower quartiles*, *whiskers*: 1.5 × interquartile range, points: outliers, n = 3).

In our initial RNAi screen, we observed the most pronounced reduction in DENV titers in ELOVL4- and FADS2-depleted cells (Fig. 1*C*). Thus, we decided to further investigate the role of ELOVL4 and FADS2 in DENV infection. We performed experiments to probe viral entry and measured viral RNA and protein levels as well as viral titers (Fig. 3*A*). First, we determined the knockdown efficiency by RT-qPCR and immunoblotting. Due to the lack of a functional ELOVL4 antibody only FADS2 knockdown levels were additionally validated by immunoblotting and confirmed a near complete loss of FADS2 protein (Fig. 3*B*). RT-qPCR showed a reduction of the respective mRNA levels to ∼20% for both shRNAs (Fig. 3*C*). Next, we analyzed if viral entry is impaired due to the depletion of FADS2 or ELOVL4. FADS2- and ELOVL4-knockdown cells were infected with a high multiplicity of infection (MOI) and internalized DENV viral RNA (vRNA) was quantified at 4 hpi. We did not observe any differences in DENV entry into target cells (Fig. 3*D*). Next, we determined vRNA copy numbers by RT-qPCR in cell lysates of DENV-infected knockdown cells. Unexpectedly, we detected a slight increase of DENV copy numbers in FADS2-knockdown cells at all time points, indicating that viral RNA replication is not or even positively affected in FADS2-depleted cells (Fig. 3*E*). In contrast, viral copy numbers in shELOVL4-expressing cells were slightly elevated at 24 hpi but did not differ from shNT control cells at 48 and 72 hpi (Fig. 3*E*). In line with increased viral RNA copies, we also observed increased levels of E and C proteins as well as NS1 in shFADS2-expressing cells, indicating that DENV RNA replication or translation is increased (Fig. 3*F*). In contrast, while the knockdown of ELOVL4 did not alter DENV copy numbers (Fig. 3*E*), it slightly decreased levels of intracellular viral proteins at 72 hpi, suggesting reduced translation or a delayed course of infection compared to shNT cells (Fig. 3*F*). In line with our initial results, both shRNAs reduced viral infectious titers but with slightly different kinetics: depletion of FADS2 continuously decreased DENV titers at all indicated time points whereas knockdown of ELOVL4 reduced infectivity only at 48 and 72 hpi (Fig. 3, *G* and *H*). Since DENV infection is cytotoxic, we next performed cytopathic effect (CPE) assays as an alternative method to assess viral replication. At 72 hpi, CPE was increased in FADS2-knockdown cells compared to shNT-transduced cells (Fig. 3*I*), correlating with the elevated replication levels in infected shFADS2 cells (Fig. 3*F*). Corresponding to the lowered viral protein levels in DENV-infected ELOVL4-knockdown cells, a slight reduction in CPE was observed compared to control cells.

### Knockdown of ELOVL4 but not FADS2 reduces DENV dsRNA foci

**Figure 4:**
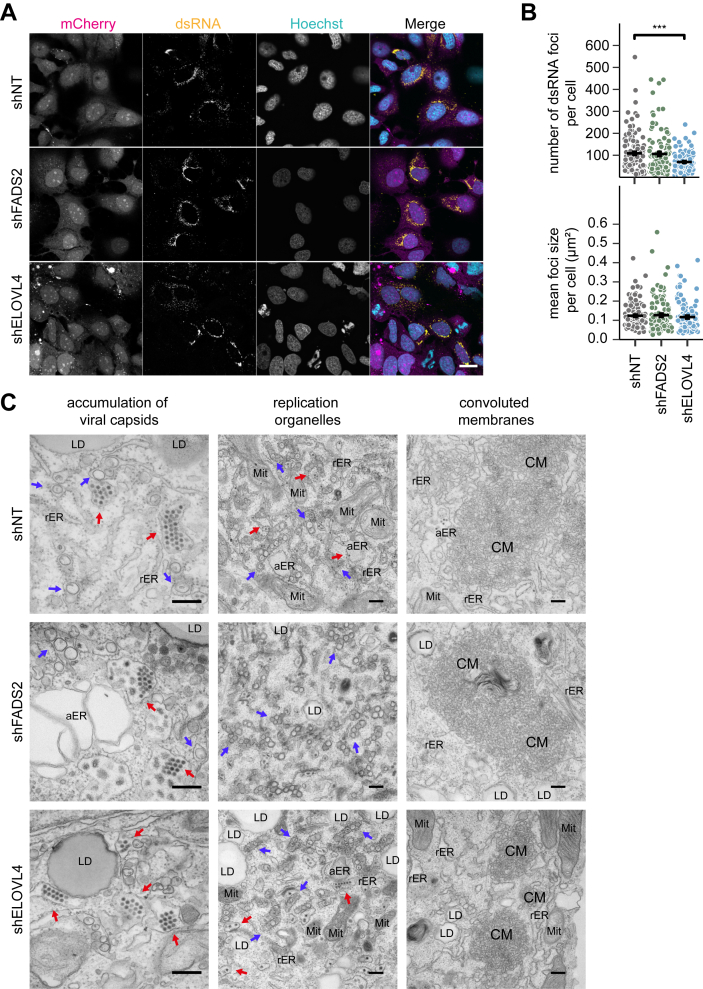
**DENV replication centers are reduced by knockdown of ELOVL4 but not FADS2.***A*, Huh7 cells were transduced with lentiviruses expressing shRNAs targeting FADS2 and ELOVL4 or a non-targeting (NT) shRNA. Four dpt, cells were infected with DENV (MOI 0.05) and fixed 48 hpi for immunofluorescence. Cells were stained with a dsRNA-specific antibody (*yellow*), nuclei were stained with Hoechst (*cyan*). mCherry (*magenta*) indicates successfully transduced cells. Scale bar 20 μM. *B*, Number and size of dsRNA foci was quantified using the particle analyzer function of Fiji Cells from 3 independent experiments were analyzed (n_shNT_ = 124, n_shELOVL4_ = 104, n_shFADS2_ = 104, Mean + SD, ∗∗∗*p* ≤ 0.001; Welch’s *t* test). *C*, Huh7 cells transduced with lentiviruses expressing shFADS2, shELOVL4, or shNT RNA were infected with DENV (MOI 0.5). 48 hpi cells were fixed and processed for EM. Shown are representative images. Scale bar 250 nm. *Arrows* indicate vesicle packets (*blue*) and virions (*red*). aER, aberrant/enlarged ER; LD, lipid droplets; Mit, mitochondrion; rER, rough ER.

To further characterize which step of DENV replication depends on FA elongation and desaturation and to address potential alterations in replication foci formation, we next analyzed the number and size of DENV dsRNA foci, representing viral RNA replication intermediates, in shELOVL4-, shFADS2-, and shNT-expressing cells by immunofluorescence staining and confocal microscopy (Fig. 4*A*). Depletion of ELOVL4 significantly reduced dsRNA foci quantity without affecting their mean size (Fig. 4*B*). In contrast, neither dsRNA foci number nor size were altered in FADS2-knockdown cells compared to controls (Fig. 4*B*).

To analyze the ultrastructure of DENV-infected cells depleted of FADS2 and ELOVL4, we performed transmission electron microscopy (TEM) (Fig. 4*C*). The membrane structures observed in DENV-infected cells are VPs (viral ROs) and unstructured convoluted membranes (CMs) (27, 28). In all conditions tested, we found VPs containing vesicles around 100 nm in size, indicating that depletion of ELOVL4 or FADS2 does not alter RO morphology (Fig. 4*C*). We also detected CMs of similar fine structure in all conditions. By semi-quantitative analysis assessing the frequency of CMs in randomly selected sections of infected cells, we observed that CMs were slightly more frequent in FADS2-knockdown cells and slightly less frequent in ELOVL4-knockdown cells compared to control cells (shNT: 35% ± 5% vs shFADS2 68% ± 4% vs shELOVL4 48% ± 2%). Further, we detected an accumulation of viral capsid structures in FADS2- and ELOVL-knockdown cells, indicating intact capsid assembly (Fig. 4*C*). Together, these data demonstrate that FADS2 is not important for viral RO formation but indicate a function in post-vRNA-replication/translation and capsid assembly steps of DENV infection, while they suggest the involvement of ELOVL4 in the formation of CMs and dsRNA foci.

### Depletion of FADS2 leads to increased extracellular viral RNA and protein levels and impairs DENV-specific infectivity

**Figure 5:**
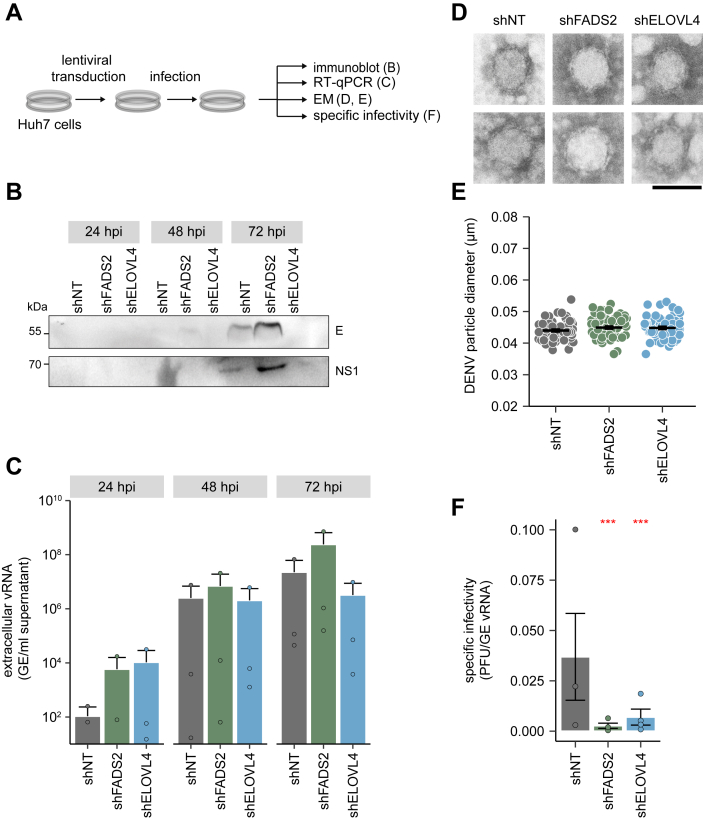
**FADS2 depletion results in elevated extracellular viral RNA and protein levels but strongly reduced specific infectivity.***A*, Huh7 knockdown cells were infected with DENV (MOI 0.05) at 4 dpt. 48 hpi, samples were harvested and processed for analysis. *B*, extracellular levels of viral E and NS1 protein were assessed by immunoblotting at indicated time points. Shown is one representative immunoblot (n = 3). *C*, extracellular virus genome copies were measured *via* RT-qPCR. Shown are virus genome equivalents (GE) per μg total cellular RNA normalized to *18S rRNA* (Mean + SD, n = 3). *D*, DENV particles secreted from knockdown or control cells were purified from supernatants harvested at 48 hpi *via* a sucrose cushion and analyzed by negative staining followed by TEM imaging. Shown are representative images of single virus particles. Scale bar 0.05 μm. *E*, DENV particle size was determined from the TEM images (n_shNT_ = 75, n_shELOVL4_ = 80, n_shFADS2_ = 76, 2 independent experiments). *F*, specific infectivity of DENV infected cells 48 hpi was calculated by the ratio of PFU/ml to DENV GE copy number (Mean + SD, n = 3–4, ∗∗∗*p* ≤ 0.001; one sample Student’s *t* test).

Knockdown of FADS2 clearly reduced DENV titers in the culture supernatant without affecting vRNA replication and translation while ELOVL4 knockdown was detrimental for replication foci formation and may additionally affect later virus replication steps. To address potential changes in virion egress, we analyzed viral protein levels as well as viral genome copies in the supernatant of DENV-infected FADS2- and ELOVL4-knockdown cells (Fig. 5*A*). Correlating with lower viral replication (Figs. 3*E* and 4*B*), depletion of ELOVL4 slightly decreased extracellular vRNA levels, and strongly reduced the amount of viral E and NS1 proteins (Fig. 5, *B* and *C*). In contrast, we did not observe differences in extracellular copy numbers in shFADS2-transduced cells and detected even higher amounts of viral proteins in the culture supernatant (Fig. 5, *B* and *C*) despite reduced infectious titers (Figs. 1 and 3).

Therefore, we next analyzed if virion morphology is affected by the knockdown of FADS2 and ELOVL4 by negative staining of concentrated DENV particles and visualization by TEM (Fig. 5*D*). Here, we did not observe any changes in the size or morphology of DENV particles, which was further substantiated by quantification of the particle size, which is equal in all conditions (Fig. 5, *D* and *E*). To determine if the specific infectivity of DENV particles produced in FADS2- and ELOVL4-knockdown cells is altered compared to the control, we determined plaque-forming units of culture supernatants as well as vRNA copy numbers and calculated the specific infectivity. Particles released from FADS2- or ELOVL4-knockdown cells showed reduced specific infectivity compared to control cells (Fig. 5*F*). Taken together the data suggest that FADS2-dependent lipids are important for the formation of infectious DENV particles.

### Inhibition of FADS2 mimics effects observed in FADS2-depleted cells

**Figure 6:**
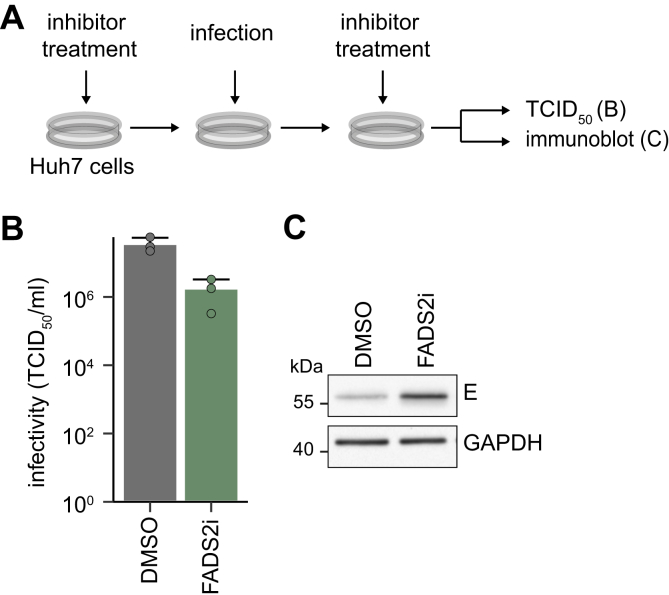
**DENV infectivity is reduced by FADS2i whereas viral protein is increased.***A*, scheme of the experiment: Huh7 cells were pretreated with 6 μm FADS2i or DMSO control for 48 h prior to infection with DENV (MOI 0.05). After removal of the virus inoculum, cells were re-treated and supernatants and cell lysates were harvested 48 hpi. *B*, infectivity was measured by TCID_50_ tirtration (Mean + SD, n = 3). *C*, cell lysates were analyzed by immunoblotting using flavivirus E antibody; GAPDH serves as loading control. Shown is one representative immunoblot (n = 3).

To confirm our results and to address if the activity of FADS2 is required for DENV replication, we next used a small-molecule inhibitor (SC-26196, FADS2i) in DENV infection. Of note, inhibitors of ELOVL4 are currently not commercially available. To mimic the shRNA-mediated knockdown, we first pretreated the cells for 2 days in media supplemented with delipidated FCS followed by infection with DENV and a second post-infection treatment again in media supplemented with delipidated FCS (Fig. 6*A*). We then analyzed viral E protein level and DENV titers 48 hpi. Correlating with the results observed in shFADS2-expressing cells, inhibition of FADS2 decreased DENV infectivity (Fig. 6*B*), indicating that FADS2 enzymatic activity is required for efficient replication. Again, we observed an increased DENV E protein level (Fig. 6*C*), confirming that FADS2 activity is required for infectious particle production and not for efficient intracellular replication and translation.

### Knockdown of FADS2 impairs DENV replication in HMC3 microglia cells

**Figure 7:**
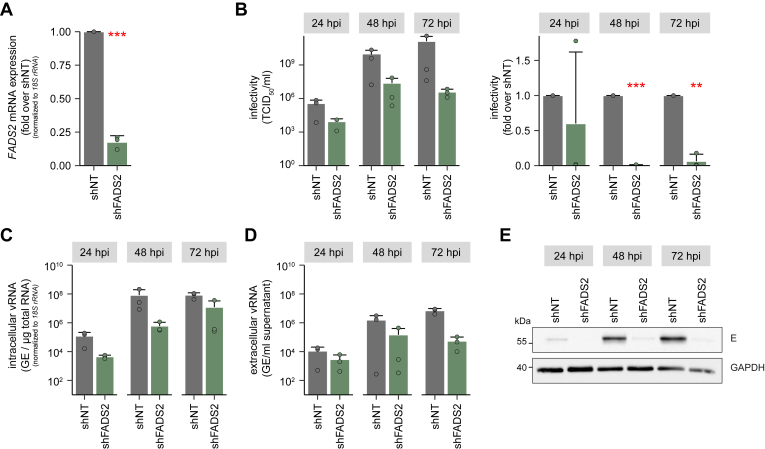
**Depletion of FADS2 reduces DENV infection in HMC3 cells.***A*, HMC3 cells were transduced with shFADS2-, shELOVL4-, or shNT-containing lentiviral particles. Knockdown efficacy was validated by RT-qPCR (Mean + SD, n = 3, ∗∗∗*p* ≤ 0.001, one sample Student’s *t* test). *B*, 4 dpt, cells were infected with DENV (MOI 0.5) and cells and supernatants were harvested at indicated time points. DENV infectious titer was determined by TCID_50_ titration and is shown as raw values or fold over shNT (Mean + SD, n = 3, ∗∗*p* ≤ 0.01, ∗∗∗*p* ≤ 0.001, one sample Student’s *t* test). *C* and *D*, virus genome copies were measured *via* RT-qPCR in lysates and culture supernatant. Shown are virus genome equivalents (GE) per μg total cellular RNA normalized to *18S rRNA* or ml culture supernatant (Mean + SD, n = 3). *E*, intracellular levels of viral E protein were assessed by immunoblotting. Shown is one representative immunoblot (n = 3).

**Figure 8:**
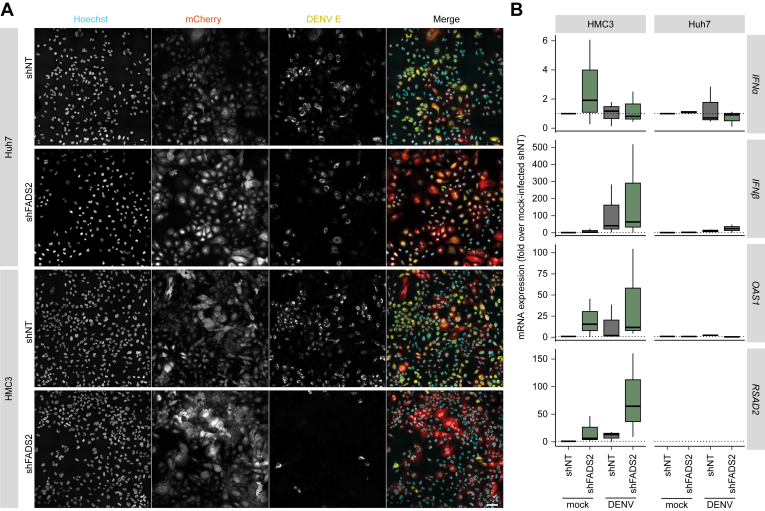
**FADS2 depletion causes a reduction of DENV infected cells *via* enhanced expression of ISGs in HMC3 cells but not in Huh7 cells**. *A*, Huh7 and HMC3 cells were transduced with shFADS2-, or shNT-containing lentiviral particles. Four dpt cells were infected with DENV (MOI 0.5) and 48 hpi fixed for immunofluorescence analysis. Cells were stained with flavivirus E antibody (*yellow*) and Hoechst (*cyan*) to visualize nuclei; mCherry (*red*) indicates successfully transduced cells. Scale bar 100 μm. *B*, Huh7 and HMC3 cells transduced with lentiviruses expressing shFADS2 or shNT were infected with DENV (MOI 0.5). 48 hpi cells were harvested for RNA extraction and analyzed by RT-qPCR (Box- and whisker plot indicates mRNA expression as fold change over mock-infected shNT-transduced cells (*center line*: median, box limits: *upper and lower quartiles*, *whiskers*: 1.5 × interquartile range, points: outliers, n = 3).

Despite DENV not being considered a neurotropic virus, encephalopathy due to DENV infiltration into patients’ brains is a common finding in severe dengue (29, 30). Therefore, we used HMC3 cells, a microglial cell line, to confirm FADS2 as a host-dependency factor in a non-cancerous immortalized cell line. Of note, the knockdown of ELOVL4 was cytotoxic for HMC3 cells (data not shown), thus we were not able to use the cells for infection experiments. First, we transduced HMC3 cells with the lentiviral constructs and confirmed the knockdown efficiency of FADS2 *via* RT-qPCR (Fig. 7*A*). Next, we analyzed infectivity in the culture supernatant and found that similar to our results in Huh7 cells, depletion of FADS2 significantly reduced DENV titers at 48 and 72 hpi (Fig. 7, *B* and *C*). At 24 hpi, titers were only marginally above the detection limit, which may be the cause for a high variance and therefore unaltered infectivity compared to the control. However, contrary to our results in Huh7 cells, knockdown of FADS2 did cause a decrease in intra- and extracellular DENV copy numbers (Fig. 7, *D* and *E*) as well as viral E protein levels (Fig. 7*F*). To further validate the differences between HMC3 and Huh7 cells, we infected and analyzed both cell types by immunofluorescence microscopy. We observed notable differences between Huh7 and HMC3 cells (Fig. 8*A*). While viral spreading was comparable between shNT and shFADS2-expressing Huh7 cells, DENV-infected foci of shFADS2-HMC3 cells were smaller and less frequent compared to control cells. This indicates that despite reduced infectious virus release, DENV is able to spread in shFADS2-transduced Huh7 cells, likely *via* cell-to-cell spread, while this route of viral spread is diminished in HMC3 cells. One major difference between Huh7 and HMC3 cells is the inability of Huh7 cells to mount an immune response to virus infection (31, 32). Interestingly, previous studies already linked FADS2 to innate immunity through the stimulator of interferon genes (STING) protein (33), a protein that is cleaved by the DENV NS2B-NS3 protease (34). Therefore, we next compared the interferon (IFN) response in DENV-infected Huh7 and HMC3 cells that do or do not express FADS2 (Fig. 8*B*). As described by others, Huh7 cells expressed only low levels of *IFNα* and *IFNß*, did not express the IFN-stimulated genes *OAS1* and *RSAD2* (viperin), and expression levels did not differ between FADS2-knockdown or control cells (Fig. 8*B*). In contrast, while we did not observe major differences in *IFNα* and *IFNß* expression levels in HMC3 cells, the expression of *OAS1* and *RSAD2* (viperin) was much higher in DENV-infected FADS2-knockdown cells than in controls, indicating that an antiviral state blocks DENV spread in FADS2-depleted HMC3 cells.

## Discussion

Flaviviruses depend on host lipid metabolism for infection. Here, we systematically investigated the role of FA elongases and desaturases in orthoflavivirus replication. In our initial shRNA-based screen, depletion of FA-elongating and desaturating enzymes only marginally altered WNV, TBEV, YFV, and ZIKV replication, indicating that these viruses can cope with altered host cell FA profiles and do not depend on lipid species harboring specific FAs. In contrast, DENV titers were strongly reduced in cells depleted of all elongases and desaturases tested, indicating that DENV needs lipids containing certain FA species to successfully replicate. Of note, the experiments were performed in the presence of FCS which may compensate partially for the lack of elongase and desaturase activity as lipids can be taken up from the medium. It is unclear to what extent compensation occurred under our experimental conditions.

We can only speculate why DENV is particularly sensitive as compared to the other flaviviruses studied. In our previous study (12), principal component analysis (PCA) of lipid remodeling in flavivirus infection demonstrated that DENV samples were most divergent from the control group, indicating more pronounced alterations in lipid composition and pointing towards differences in lipid requirement. In addition, in entry and viral RNA replication assays, DENV was less efficient than the other flaviviruses, which may render DENV more susceptible to interference with lipid remodeling. In the present study, we performed an analysis of enriched lipid annotation terms, which indicated that DENV infection led to an upregulation of terms that are linked to longer and more desaturated FAs. WNV-infected cells did show a similar profile of enriched terms, but WNV may be less sensitive to blocking FA elongation and desaturation due to its very efficient infection kinetics in Huh7 cells. In addition to unique lipid metabolic changes, DENV virions were shown to be more temperature-sensitive compared to ZIKV and WNV virions (35, 36, 37) and in our hands, DENV particles are less stable than the particles of the other flaviviruses we are working with. This may explain why interference with FA elongation and desaturation affects the infectivity of DENV particles more than other flaviviruses.

17β-Hydroxysteroid dehydrogenase type 12 (17β-HSD12), the 3-ketoacyl-CoA reductase of long-chain FA synthesis, was suggested to be a host co-factor for DENV and ZIKV and the related hepacivirus HCV, thereby supporting the hypothesis that DENV depends on ULCFAs (38, 39). Interestingly, in infected mosquito cells, DENV infection, but not YFV or WNV infection, led to downregulation of AAEL012402 expression, which encodes an elongase enzyme (40). In our experiments in human hepatoma cells, the expression of elongases and desaturases was only marginally altered after infection with DENV.

In RNAi experiments, we observed a strong reduction of DENV infectious titers in all cells harboring shRNAs targeting elongases and desaturases. This is in contrast to a previous study showing no significant reduction of DENV infectious viral particles using siRNA-mediated knockdown for ELOVL2, ELOVL5, ELOVL6, SCD5, FADS1, and FADS2 in Huh7 cells (21). These diverging results might be due to the use of transient siRNA *versus* a stable lentiviral shRNA system to downregulate the respective enzymes. In addition, no validation of siRNA-mediated knockdown efficacy was reported (21) while we previously validated a robust knockdown using the described shRNAs (23). The same study revealed that SCD, the rate-limiting enzyme in MUFA synthesis, is an essential host factor for DENV replication and infectious particle production (21). This is in line with our observation that DENV infectious titers dropped in SCD-depleted cells. Of note, SCD was previously reported to be marginally but consistently upregulated during DENV-2 infection in HEK293 T cells, but first up- and then downregulated after infection in HepG2 cells (41), similar to what we observed in Huh7 cells (Fig. 2*B*).

Among all elongases analyzed in our initial shRNA-screen, depletion of ELOLV4 most significantly reduced DENV infectious titers and subsequent experiments confirmed a significant reduction of vRNA titers as well as viral protein levels. Correspondingly, ELOVL4 depletion decreased dsRNA formation and foci number. As FA carbon chain length and desaturation can influence membrane fluidity and curvature (42, 43), we examined RO morphology. DENV-infected shELOVL4 knockdown cells had similar RO morphology compared to shNT cells, suggesting that ROs can still be formed but replication might be less efficient. We have previously shown that the elongases ELOVL2, ELOVL4, ELOVL6, and ELOVL7 are important for HCV RNA replication but not for virion production (23). ELOVL4 and ELOVL7 are also known to be essential for replication of the hepatitis A (HAV) genome (44, 45). In contrast, human cytomegalovirus (HMCV) infection increased expression of multiple long-chain acyl-CoA synthetases and FA elongases and their inhibition led to virus particles with reduced infectivity (45). Cells infected with HCMV upregulate elongases, especially ELOVL7, to increase the abundance of long-chain FAs, which are then incorporated into the viral envelope (46). We identified fewer viral proteins in the supernatant of shELOVL4 knockdown cells. Additionally, isolated particles revealed no morphological changes compared to shNT cells but the specific infectivity was decreased in comparison to the virions released from control cells. This indicates that ELOVL4 is important for both DENV replication and virion production.

Regarding FADS2, the most prominent FA desaturase in our screen, knockdown, and pharmacological inhibition in Huh7 cells significantly decreased DENV infectious titers. Unexpectedly, loss of FADS2 led to increased intra- and extracellular viral protein levels, marginally increased vRNA levels, and intact vRNA replication structures. DENV particles produced by shFADS2 cells were also structurally comparable to particles produced by shNT cells, indicating that the reduced infectivity was not due to defective virion formation, but a result of the strongly reduced specific infectivity of the supernatant. In previous work, we showed that HCV infection increased PUFA levels, and FADS2 activity is needed for efficient HCV particle production without affecting viral RNA replication (23). Conversely, FADS2 was also demonstrated to act as a proviral factor promoting HCV replication of different HCV strains whose replicase complex is sensitive to ferroptosis (47). Regarding DENV, our data indicate a role for FADS2 activity in promoting virion infectivity. A lipid bilayer derived from host cell membranes surrounds the flavivirus nucleocapsid core (48). The composition of this bilayer is not fully understood, but it is estimated that there are approximately 8000 lipid molecules in the DENV envelope, consisting mainly of phosphatidylcholine, phosphatidylethanolamine, phosphatidylserine, sphingomyelin, and ceramides (49). It has also been suggested that the incorporation of lipids into the DENV virion varies between cell types (48). For WNV, another important member of the orthoflavivirus family, sphingolipids are enriched in the envelope (50). Pharmacological inhibition of neutral sphingomyelinase altered WNV morphogenesis, supporting the hypothesis that alteration of host cell lipid composition can directly influence virus envelope formation and thus particle infectivity (50). The reduced DENV infectivity, therefore, could be due to changes in the lipid composition of the viral envelope due to a lack of FADS2 activity. It remains to be elucidated how FADS2 activity impacts DENV lipid composition.

Further characterization of FADS2 reliance in HMC3 cells revealed a strong reduction in infectious titers. This indicates that the dependency of DENV on FADS2 is not restricted to a single cell type. Interestingly, the effects in HMC3 microglial cells were even stronger, likely affecting earlier events in infection. Previous studies demonstrated an interaction between FADS2 and STING, a protein that is involved in cytosolic nucleic acid-associated inflammatory responses. FADS2 desaturase activity inhibited STING and enhanced infection of a STING-dependent virus (33). In addition, STING is a target of DENV NS2B-NS3 protease (34). Here, we show that depletion of FADS2 strongly reduced DENV infection in HMC3 cells, which, in contrast to Huh7 cells, are immune-competent. Our data indicate that the depletion of FADS2 increases the IFN response in DENV infection, adding a second mechanism to how FADS2 influences DENV infection.

Taken together our results demonstrate that DENV is an exception among the analyzed orthoflaviviruses as it critically depends on FA elongation and desaturation. Future directions should focus on investigating the molecular functions of the specific lipids in DENV infection.

## Experimental procedures

### Cell lines, culture conditions, and viability assays

Huh7 was provided by R. Bartenschlager, HEK293T and Vero E6 from the American Type Culture Collection, and BHK21 from C. Munoz-Fontela. Cells were cultivated under standard cell culture conditions in high glucose DMEM supplemented with 10% FCS (Gibco), or delipidated FCS (PAN-Biotech), GlutaMax, and with or without Pen/Strep (Gibco). Cell viability was analyzed by CellTiter96 Aqueous One Solution Reagent (Promega). Calcium phosphate precipitation was used for transfection for lentivirus production.

### Plasmids

The plasmids used for generating viral stocks were described previously: pFK-DVs encoding DENV-2 16,681 (51), pACNR-FLYFV-17Da encoding YFV-17D (52, 53), pWN-AB and pWN-CG encoding WNV NY99 (54), pJW231 and pJW232 encoding ZIKV PRVABC59 (55), and pTNd/c encoding TBEV Neudörfl strain (56). Lentiviral shRNA constructs targeting elongases and desaturases (shELOVL1, shELOVL2, shELOVL3, shELOVL4, shELOVL5, shELOVL6, shSCD, shSCD5, shFADS1, shFADS2, shNT) were described previously (23).

### Antibodies and reagents

The following antibodies were obtained commercially: FADS2 specific antibody (Abcam), orthoflavivirus group antigen D1-4G2-4-15 (Novus `Biologicals), dengue virus capsid protein (GeneTex), flavivirus NS1 (abcam), GAPDH (Santa Cruz), Alexa 488-conjugated IgG (donkey, (H+L), Invitrogen), HRP-labeled secondary antibodies (Jackson Laboratories). FADS2 antibody (Abcam) was used for immunoblot and validated using lysates of shFADS2-expressing cells compared to shNT and orthoflavivirus group antigen D1-4G2-4-15 was validated using infected and uninfected cell lysates and only showed specific signal in infected cell lysates. Nuclei are stained with Hoechst 33,342 (H1399) (Invitrogen). SC-26196 (FADS2i, 4 mM stock in DMSO, f.c. 6 μM) was purchased from Biotechne. If not noted otherwise enzymes for molecular cloning were bought from New England Biolabs, cell culture reagents from Gibco/Life Technologies, and fine chemicals from AppliChem or Sigma.

### *In vitro* transcription of flavivirus RNA, production of virus stocks, and infection

Plasmids were linearized and purified by phenol-chloroform extraction to prepare DENV, TBEV, and YFV-17D viral stocks, as previously described (12). In detail, for WNV and ZIKV, we used a two-plasmid system with an overlap PCR strategy with the following primers: ZIKV pJW231 BamHI fw TAGGATCCTAATACGACTCACTATAG; ZIKV Conserved 3499Rev, GCCTTATCTCCATTCCATACCA; ZIKV pJW232 ApaLI fw AGGGAGTGCACAATGCCCCCA; ZIKV pJW232 EcoRI rev CTAGAATTCGCCCTTGCTCCG; WNV AB fw CATCTCAGCTCTTGCCGGCTGATGTCTATGGCAC; WNV AB rev ACGCGTAAATTTAATACGACT; WNV CG fw TCTAGAGATCCTGTGTTCTC; WNV CG rev GCCGGCAAGAGCTGAGATGT. The overlap PCR products were purified by gel extraction.

*In vitro* transcription was performed with the MegaScript SP6 Transcription Kit and Cap Analog (m7G(5′)ppp(5′)G) (ThermoFisher) for DENV and YFV-17D, or HiScribe T7 ARCA mRNA Kit (NEB) for all other viruses. Virus stocks were produced in Vero E6 cells with exception of TBEV, which were produced in BHK-21 cells. For electroporation, 4 × 10^6^ cells were washed in Opti-MEM (Life Technologies) and resuspended in 400 μl cytomix buffer (120 mM KCl, 5 mM MgCl_2_, 0.15 mM CaCl_2_, 2 mM EGTA, 1.9 mM ATP, 4.7 mM GSH, 25 mM HEPES, 10 mM potassium phosphate buffer, pH 7.6). 5 μg of *in vitro* transcribed orthoflavivirus RNA was added to the cell suspension and pulsed at 260 V and 950 μF using the Gene Pulser II (Biorad). For P1 virus stock was produced by infection of naïve BHK21 or Vero E6 cells with P0 virus stock. For infection of Huh7, virus stocks were diluted in DMEM containing 10% FCS and added to cells for 1 h if not stated otherwise.

### Determination of viral titers (TCID_50_)

For virus stocks (except TBEV) the viral titers were determined using Huh7 cells *via* tissue culture infectious dose titration (TCID_50_). BHK21 cells were used to titrate supernatants of infection experiments and for the production and titration of TBEV viral stock. Cells were seeded in 96-well plates 1 day prior to infection in a serial dilution of 1:10^1^ to 1:10^7^ or 1:10^11^. Five days after titration, cells were fixed and stained with crystal violet. For the calculation of TCID_50_/ml, we used the Reed and Muench calculator from B. Lindenbach (57).

### Lentivirus production and transduction

Lentiviral particles were produced as described previously (58). To generate lentiviral particles, 293T cells were co-transfected with the pSicoR-MS1 shRNA constructs, a packaging construct (pCMVΔR8.91), and a construct expressing the glycoprotein of vesicular stomatitis virus (VSV-G) (pMD.G) followed by concentration *via* ultracentrifugation. Transduction of lentiviral plasmids was performed in DMEM supplemented with 4 μg/ml polybrene (Sigma). Titration of Lentiviral stocks was performed using flow cytometry (Guava easyCyte HT Cytometer) or microscopy.

### CPE assay

For CPE reduction assay, the shRNA-transduced Huh7 cells were infected with DENV (MOI 0.5). At 3 dpi the cells were fixed and CPE was visualized by crystal violet staining. The scanning of the plates was performed on a ChemiDoc imaging system (BioRad) and CPE was quantified using Fiji/Image J.

### Plaque assay

For plaque assays, Vero E6 cells were seeded at 90 to 95% confluence 1 day prior to infection. Tenfold serial dilutions of the virus-containing supernatant were added to each well. Cells were incubated at 37 °C with 5% CO₂ for 1 h and shaken every 15 min to ensure equal infection of the cell monolayer. The inoculum was removed and the cells were overlaid with a 1.25% Avicel in 1 × MEM mixture. The cells were incubated at 37 °C with 5% CO₂ for 5 days, then fixed and stained with crystal violet to visualize plaque formation.

### Immunoblot analysis

For immunoblotting cells were lysed 1 h on ice in RIPA buffer (150 mM NaCl, 50 mM Tris-HCl pH 7.6, 1% NP-40, 0.5% sodium deoxycholate, 5 mM EDTA, protease inhibitor cocktail (Sigma), PMSF (AppliChem)). Lysates were clarified by centrifugation and subjected to SDS-PAGE. For extracellular protein analysis, culture supernatant was inactivated with 6× Laemmli buffer and heated for 10 min at 70 °C prior to loading. Proteins were transferred to nitrocellulose membranes. After blocking in 5% skim milk/1 × TBS-T, membranes were incubated with primary followed by staining with HRP-coupled secondary antibodies to detect chemiluminescence with Lumi-Light substrate (Roche), Imobilon Forte (Sigma-Aldrich), or SuperSignal West Femto (Thermo Fisher) on a ChemiDoc imaging system (BioRad). For the quantification of the band signal intensities the Image Lab (BioRad) software was used.

### Immunofluorescence microscopy

For microscopy, cells were seeded on coverslips 1 day prior to infection and fixed 48 hpi in 4% paraformaldehyde. Cells were then permeabilized in 0.1% triton-X-100 in PBS for 7 min and blocking was performed for 1 h in blocking solution (5% BSA, 1% fish skin gelatin, 50 mM Tris in PBS). After staining with primary antibodies overnight and incubation with Alexa Fluor-conjugated secondary antibodies for 1 h, coverslips were embedded in Mowiol. Samples were analyzed on a Leica TCS SP5 II confocal laser scanning microscope and on a Leica Stellaris 8 confocal laser scanning microscope. dsRNA Foci were quantified using the particle analyzer function of Fiji.

### RNA isolation and quantitative RT-PCR

Viral RNA of infected culture supernatants was isolated with Nucleo Spin RNA Virus Kit (Machery Nagel) and total cellular RNA using TriReagent (Sigma-Aldrich) according to manufacturers’ protocol. Cellular DNA was digested with rDNaseI using the TURBO DNA-free DNase kit (Ambion). For reverse transcription into cDNA, we used random hexamer primers (Qiagen), SuperScript III reverse transcriptase (Thermo Fisher), and RNaseOut (Thermo Fisher). qPCR was performed with Luna Universal qPCR master mix (New England Biolabs). For quantitative PCR, we used the following primers: FADS2 fw TGACCGCAAGGTTTACAACAT, FADS2 rev AGGCATCCGTTGCATCTTCTC, ELOVL4 fw GAGCCGGGTAGTGTCCTAAAC, ELOVL4 rev CACACGCTTATCTGCGATGG, 18S rRNA fw GTAACCCGTTGAACCCCATT, 18S rRNA rev CCATCCAATCGGTAGTAGCG DENV fw GCAGAAACACAACATGGAACRATAGT, DENV rev TGATGTAGCTGTCTCCRAATGG, OAS1 fw GCCCTGGGTCAGTTGACTGG, OAS1 rev TGAAGCAGGTGGAGAACTCGC, RSDA2 sense qPCR TTGGACATTCTCGCTATCTCCT RSDA2 as qPCR AGTGCTTTGATCTGTTCCGTC, IFNα sense TCCATGAGATGATCCAGCAG IFNαA as ATTTCTGCTCTGACAACCTCC IFNβB qPCR sense GCTTGGATTCCTACAAAGAAGCA, IFNβB qPCR as ATAGATGGTCAATGCGGCGTC.

### Electron microscopy

For negative staining of virions, the purified virus particles were adhered to formvar-coated TEM grids and treated with 2% phosphotungstic acid. For ultrastructural analyses, cells were grown on sapphire discs (Engineering Office M. Wohlwend GmbH). The cells were fixed for 1 h in 0.1 M Na-cacodylate buffer pH 7.2 containing EM-grade 2.5% glutaraldehyde and 2% formaldehyde, followed by washing and incubation in 1% osmium tetroxide containing 1.5% ferricyanide for 1 h in the same buffer. The cells were incubated in 2% uranyl acetate in water overnight, dehydrated in a series of graded ethanol solutions into acetone, and subsequently infiltrated with resin (EMbed-812 Kit with BDMA), and polymerized at 60 °C for 48 h. The sapphire disc was removed to expose the cells at the blockface. Ultrathin sections were mounted on formvar-coated grids and double-stained with 2% uranyl acetate and 3% lead citrate. Samples were imaged at an acceleration voltage of 120 kV in a JEOL JEM-1400 TEM equipped with a TemCam-F416 camera (TVIPS; unless otherwise mentioned, the materials are from Science Services).

### Quantification and statistical analysis

R and RStudio were used to visualize data and for statistical analysis (59, 60). Statistical analysis was performed using unpaired two-tailed Welch's unequal variances *t* test, Mann-Wittney-U-test, or, in case of normalized data, one sample Student’s *t* test, and adjusted for multiple comparisons with the false discovery rate (FDR) as indicated in the figure legends. Sample sizes (n) represent independent experiments, if not stated otherwise. The data was analyzed and visualized using several packages available for R, namely dplyr (version 1.1.4) (61), tidyr (62), (version 1.3.0), ggplot2 (version 3.4.4) (63), pheatmap (version 1.0.12) (64), cowplot (version 1.1.1) (65), ggrepel (version 0.9.4) (66), purrr (version 1.0.2) (67), coin (version 1.4–3) (68), as well as LION/web (version v.2023.04.14) (http://www.lipidontology.com) with missing data imputed with MetImp 1.2 (https://metabolomics.cc.hawaii.edu/software/MetImp/) (69) using MCAR/MAR method RF.

## Data availability

All data are available within the article or its supplementary materials.

## Supporting information

This article contains supporting information (12).

## Conflicts of interest

The authors declare that they have no conflicts of interest with the contents of this article.
