# Supplementary material for: Dengue virus is particularly sensitive to interference with long-chain fatty acid elongation and desaturation
Source: J Biol Chem. 2025 Jan 23;301(3):108222. doi: 10.1016/j.jbc.2025.108222 (PMC11908578; doi:10.1016/j.jbc.2025.108222)
Supplement: Supplemental information [file mmc1.pdf]

# Dengue virus is particularly sensitive to interference with long chain fatty acid elongation and desaturation

Julia Hehner[1], Lisa Ludenia[1], Laura Bierau[1], Anja Schöbel[1], Martin Schauflinger[1], Yvonne F. Grande[1], Dominik Schwudke[2,3,4], and Eva Herker[1]

## Description of Supporting Information

### Table S1

Title: Lipidomics data of lipid species

Description: Amount of all lipids identified in DENV-infected cells and mock controls 48 hpi in 3 independent experiments in mol%. Input data for imputing missing values and subsequent LION/web analysis shown in Figure 2. This dataset was previously published (1).

### Table S2

Title: LION/web analysis

Description: Results of the LION/web analysis displayed in Figure 2.

### References

1. Hehner, J., Schneider, L., Woitalla, A., Ott, B., Vu, K. C. T., Schobel, A. *et al.* (2024) Glycerophospholipid remodeling is critical for orthoflavivirus infection Nat Commun **15**, 8683 10.1038/s41467-024-52979-y
